# Early detection of Alzheimer's disease using deep learning methods

**DOI:** 10.1002/alz.70175

**Published:** 2025-05-12

**Authors:** Anthony Chidubem Mmadumbu, Faisal Saeed, Fuad Ghaleb, Sultan Noman Qasem

**Affiliations:** ^1^ College of Computing Birmingham City University Birmingham UK; ^2^ Computer Science Department College of Computer and Information Sciences Imam Mohammad Ibn Saud Islamic University (IMSIU) Riyadh Saudi Arabia; ^3^ King Salman Center for Disability Research Riyadh Saudi Arabia

**Keywords:** Alzheimer's disease, artificial intelligence, biomarker, cognitive test, data processing, early detection, feedforward neural network, hybrid models, long short‐term memory, machine learning, MobileNetV2, neural network, ResNet50

## Abstract

**INTRODUCTION:**

Alzheimer's disease (AD), a leading cause of dementia, requires early detection for effective intervention. This study employs AI to analyze multimodal datasets, including clinical, biomarker, and neuroimaging data, using hybrid deep learning frameworks to improve predictive accuracy.

**METHODS:**

A novel framework was developed, including trained models for structured data and magnetic resonance images. The structured data model used a long short‐term memory (LSTM) for temporal dependencies and a feedforward neural network (FNN) for static patterns. The MRI‐based model employed ResNet50 and MobileNetV2 to extract spatial features. Models were applied on National Alzheimer's Coordinating Centre (NACC) and Alzheimer's Disease Neuroimaging Initiative (ADNI) datasets and compared to previous works.

**RESULTS:**

The MRI‐based model achieved 96.19% accuracy on the ADNI dataset, while the hybrid model attained 99.82% accuracy on NACC dataset.

**DISCUSSION:**

This study highlights hybrid AI models' potential in AD detection, enabling earlier interventions and improved detection outcomes.

**Highlights:**

- AI models were explored for early AD detection using NACC and ADNI datasets.
- Achieved high accuracy with LSTM on NACC data, showing potential for early AD diagnosis.
- Evaluated transfer learning models (MobileNetV2, ResNet‐50) to address data limitations.
- A method is proposed for the careful validation of transfer learning models in medical brain diagnostics.

## 1 BACKGROUND

The slow onset, subtle symptoms, and gradual cognitive decline characteristic of Alzheimer's disease (AD) make it one of the most challenging neurodegenerative conditions to detect early, which hinders swift and accurate diagnosis.^1^ AD is one of the leading causes of dementia in the United Kingdom and many other countries worldwide.^1^

Novel detection methods are increasingly necessary, as traditional approaches remain costly and difficult to access and are often imprecise for early‐stage identification.^2^ This study explores the transformative capabilities of advanced machine learning (ML) models for structured and imaging data modalities. Specifically, two independent models are proposed: a hybrid model combining long short‐term memory (LSTM) and feedforward neural networks (FNNs) for structured data and another integrating ResNet50 and MobileNetV2 for MRI scan analysis. These models, while developed independently, are conceptualized to function as components of a healthcare system that supports clinical decision‐making by providing complementary predictions from structured or image data modalities. This approach provides flexibility for decision‐makers, such as healthcare professionals, to leverage insights from structured data (e.g., questionnaires) or medical images (such as MRI) for improved diagnostic accuracy.

While advancements in cognitive tests and biomarkers have not been so promising in the context of early diagnosis,^3^, ^4^ ML and deep learning have leveraged medical imaging to help identify early signs of AD, brain tumors, and Parkinson's disease.^5^ The National Alzheimer's Coordinating Centre (NACC) has been at the forefront of these synergetic successes as datasets from the organization have played a vital role in leveraging AI success in the field; a good example is a 2024 study by Alatrany et al.^6^ that leveraged ML to enhance early AD diagnosis. The study used a support vector machine (SVM) model to achieve high performance of 90.5% accuracy for multiclass classification.^6^ Another notable study^7^ utilized MRI scans and clinical data to identify different stages of AD and its progression from mild cognitive impairment to AD, achieving an accuracy of 95.52%.

To build on these advancements, this research developed two complementary models. LSTM leveraged the temporal dependencies in the structured dataset critical to understanding AD progression^8^; these subsets of the datasets were regarded as the sequential features because they capture changes observed in the participants over time. Similarly, non‐sequential features such as demographic and static insights were processed through a FNN.^9^ For the MRI scans, the analysis involved preprocessing the images by resizing them to a uniform resolution, followed by normalization and data augmentation techniques such as rotation, flipping, and zooming. ResNet50 captured high‐level structural patterns, while MobileNetV2 leveraged its computational efficiency to capture subtle regional variations.

**RESEARCH IN CONTEXT**

1. **Systematic review**: Previous studies demonstrated the potential of deep learning models, such as LSTM for structured data and CNNs like ResNet for medical image analysis. However, achieving high accuracy and generalizability remains challenging, particularly with limited or geographically constrained datasets. Research highlights the need for multimodal biomarkers, including MRI scans, alongside cognitive assessments to improve AD classification. Conventional ML methods, such as SVMs, rely on manual feature selection, potentially limiting model efficiency.
2. **Interpretation**: Our study enhances AD detection by independently evaluating structured and MRI‐based models using the diverse NACC and ADNI datasets. The hybrid LSTM‐FNN model captured temporal dependencies and static correlations, achieving 99.82% accuracy on structured data, while transfer learning models ResNet50 and MobileNetV2 identified spatial patterns in MRI images with 96.19% accuracy. Independent training of these models provides flexibility in clinical applications where structured or imaging data may be available separately.
3. **Future directions**: Future research should explore model validation across diverse populations, uncertainty estimation for clinical reliability, computational optimizations for resource‐limited settings, real‐time disease monitoring, and the integration of both models into a unified clinical decision‐support system.

To develop an optimal model using the structured dataset, rigorous feature selection methods were implemented to handle issues like model training redundancy. The feature selection methods applied are Sequential Feature Detachment (SFD) for sequential data^10^, ^11^ and correlation‐based pruning for non‐sequential features.^12^ These efforts highlight the importance of robust feature engineering in enabling each model to specialize in its respective domain.

Existing studies^13^, ^14^, ^15^ underscore the potential of AI in AD diagnosis but highlight significant limitations due to undiversified datasets. These limitations hinder model generalization and lead to poor performance when models are tested on datasets different from their training data.^16^ To address this issue, this study has employed datasets of different sources (multimodal) structured by NACC and 26 MRI scans from the Alzheimer's Disease Neuroimaging Initiative (ADNI) to train, evaluate, and test both models’ performance and improve generalization.

**FIGURE 1:**
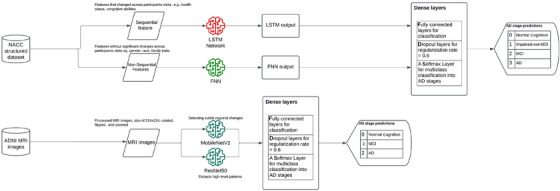
Architecture of proposed hybrid model combining long short‐term memory and feedforward neural network for structured data and MobileNetV2 and ResNet50 for MRI image data. Outputs are classified into Alzheimer's disease stages using dense layers and a Softmax layer.

In recent times, researchers have identified that while integrating multimodal data for improved accuracy and generalization is essential,^17^ the approach presents challenges in reconciling various data types.^18^ This is another area this research has investigated by applying different innovative approaches to handle these different data modalities. Figure 1 provides a visual representation of the innovative structural design of the proposed model.

A summary of the performance of the existing methods is presented in Table 1.

**TABLE 1 alz70175-tbl-0001:** Comparison of related works, highlighting algorithms, strengths, weaknesses, architectures, features used, and evaluation metrics.

Number	Algorithms	Strength	Weakness	Architecture	Feature Used	Research Name	Evaluation Metrics
1	Hybrid LSTM and feedforward	‐ Combines sequential and non‐sequential data ‐ Captures both temporal and static patterns with statistical methods like rolling window and sequence length across hashed participant IDs ‐ High accuracy and robustness	‐ Computationally intensive ‐ Requires careful feature selection	Parallel LSTM and feedforward network (feature fusion)	621 sequential features (90th percentile variability), 400 non‐sequential features	Current research	Accuracy: 99.82% Precision: 99.82% Recall: 99.82% F1 Score: 99.82%
2	LSTM‐only model	‐ Effective for learning sequential patterns ‐ Good performance with time‐series data ‐ Incorporates hashed participant ID for tracking	‐ Lacks integration of non‐sequential features ‐ Slightly lower performance compared to hybrid models	Two LSTM Layers with time‐series feature engineering	Features selected by decision trees and domain knowledge research	Current research (second experiment)	Accuracy: 97.68% Precision: 97.85% Recall: 97.68% F1 Score: 97.71%
3	Multimodal multitask deep learning model	‐ Integrates multimodal time series data and background knowledge ‐ Jointly predicts progression and cognitive scores	‐ High computational cost ‐ Complex architecture prone to overfitting	Stacked CNN‐BiLSTM with feedforward network	Multimodal data (five types of time‐series data + background knowledge)	El‐Sappagh et al.^20^	Accuracy: 97.84% Precision: not provided Recall: not provided F1 score: not provided
4	Hybrid model	‐ Effective for sequential data ‐ Captures long‐term dependencies ‐ Versatile for diverse tasks	‐ Prone to overfitting with small datasets ‐ Computationally expensive	Stacked CNN and LSTM	Single modality (MRI scan)	Saravanakumar and Saravanan^21^	Accuracy: 98.67% Precision: 98.86%
5	Deep learning	‐ Learns hierarchical representations automatically ‐ Excellent for large, complex datasets	‐ Large, labeled data is needed for training ‐ High computational demands ‐ Prone to overfitting	Transfer learning CNN	Single modality (MRI scans)	Prakash et al.^21^	Accuracy: 98.37%
6	Transfer learning	‐ Leverages pretrained models for performance improvement	‐ Generalizability issues with limited modalities ‐ Performance restricted to specific datasets	Residual neural networks	Single modality (resting‐state fMRI)	Ramzan et al.^22^	Accuracy: 97.92%

## 2 METHODS

This study utilized the NACC investigator_nacc63.csv dataset, acquired on December 15, 2023 (data request number 11,715). This dataset, comprising demographic, clinical, and cognitive data, serves as a robust foundation for AD research. To complement this, 26 prelabeled MRI images from the ADNI MRI databank were included to independently evaluate a deep learning‐based image analysis model. These MRI images are classified into three Alzheimer's progression stages.

The study followed the Cross‐Industry Standard Process for Data Mining (CRISP‐DM) to guide data preparation, modeling, and evaluation.

### 2.1 Data preprocessing

Data preprocessing was tailored separately for structured and image datasets to suit their unique characteristics.

### 2.2 Structured data

**FIGURE 2:**
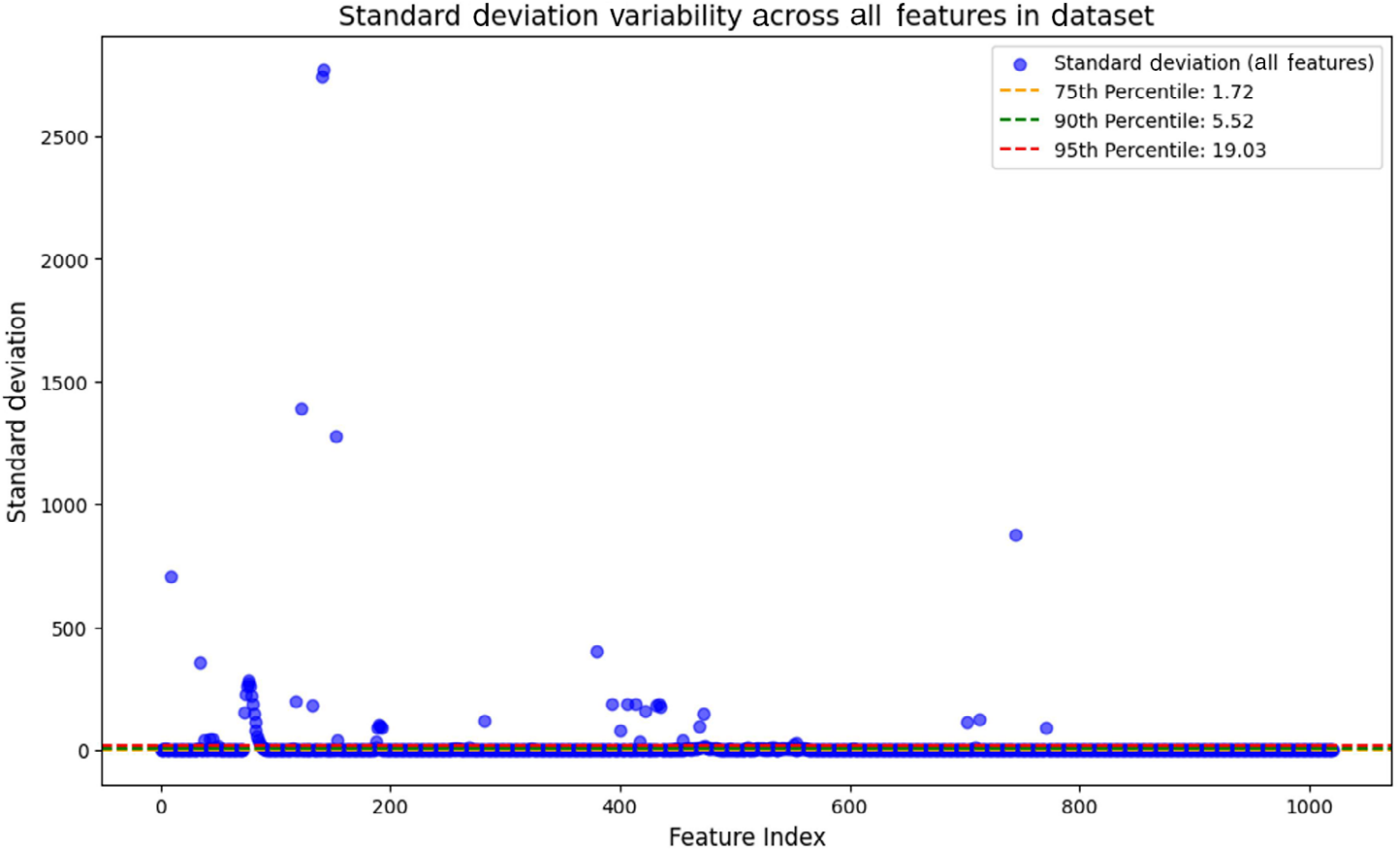
Scatter plot showing standard deviation of all features in dataset, with reference lines indicating 75th, 90th, and 95th percentile thresholds.

The structured dataset was split into sequential and non‐sequential features. For the sequential features a scatter plot of feature[Table alz70175-tbl-0001] variability (Figure 2) was used to select features in the 90th percentile of standard deviation, capturing high‐variability features for the LSTM model.

Explanation of visual elements in the scatter plot:

1. Feature index (*x*‐axis): This represents each feature in the dataset as an individual point.
2. Standard deviation (*y*‐axis): This reflects the variability of each feature across participants.
3. Line colors

- Orange: 75th percentile, showing where 75% of features fall below.
- Green: 90th percentile, the selection threshold for high‐variability features.
- Red: 95th percentile, highlighting the top 5% of variability.
- Blue: Mean standard deviation, the average variability of all features.

4 Blue Dots: Each dot corresponds to a feature plotted by its index and variability. The plot shows the variability of many features are close, so we decided to select the 90th percentile (top 10%) to reduce redundancy in the LSTM learning process and move the reset to FNN after correlation‐based pruning redundancy in FNN.

Finally, the sequential features were sorted by participant ID and visit time to maintain temporal order. The non‐sequential features are the features below the 90th percentile threshold, which underwent correlation‐based pruning to eliminate redundancies.^12^ Strongly correlated features (correlation > 0.85) were removed, reducing the feature set from 429 to 268 for training in a FNN.

### 2.3 Standardization and normalization

1. Sequential features were standardized using z‐score normalization.
2. Non‐sequential features were scaled with min‐max normalization.
3. Categorical variables were one‐hot encoded.

The target variable, representing AD progression, was dynamically encoded into four classes:

- **0**: Normal cognition (NC)
- **1**: Impaired‐not‐MCI (I‐n‐MCI)
- **2**: Mild cognitive impairment (MCI)
- **3**: Alzheimer's disease (AD)

### 2.4 Dataset splitting

To prevent data leakage, the structured dataset was split into training, validation, and test sets by participant ID, ensuring that no data from the same individual appeared in multiple sets.

### 2.5 Image data

MRI images were resized to 224 × 224 pixels and normalized for uniform scaling. To enhance generalizability, data augmentation techniques, including rotation, flipping, and zooming, were applied.

### 2.6 Feature extraction

The pretrained deep learning models, ResNet50 and MobileNetV2, were employed to extract features from the MRI images:

1. ResNet50 captured high‐level spatial patterns indicative of AD progression, such as cortical thinning and atrophy.
2. MobileNetV2 focused on subtle regional changes, including hippocampal variations, with computational efficiency.

Each model produced independent feature representations of the images, which were used to train and evaluate their respective classification networks.

### 2.7 Model architectures

#### 2.7.1 Structured data models

1. LSTM for sequential features

Sequential features were processed using a two‐layer LSTM network with 32 and 16 units, respectively. Rectified linear unit (ReLU) activations were applied to mitigate vanishing/exploding gradient issues. Dropout layers (rate = 0.2) were used to reduce overfitting.

2. FNN for non‐sequential features

Non‐sequential features were processed through a FNN with two fully connected layers (32 neurons each, ReLU activation).

Batch normalization accelerated convergence and stabilized training.

### 2.8 MRI image models

Two separate models were trained using the features extracted by ResNet50 and MobileNetV2.

Each model included dense layers to map the extracted features to the four AD progression classes. A Softmax layer was used for multiclass classification, outputting probabilities for each progression stage.

### 2.9 Technical implementation

All models were implemented using Python with TensorFlow and Keras frameworks. NumPy and Pandas libraries facilitated data handling, while Matplotlib and Seaborn supported visualizations.

The “categorical crossentropy loss” function was used for multiclass classification.

The “Adam optimizer” with an initial learning rate of 0.001 was employed.

Hyperparameters, including the number of LSTM units, learning rates, and dropout rates, were optimized using grid search.

### 2.10 Training and validation

Models were trained independently for structured and MRI data. Training and validation metrics, including accuracy, loss, precision, recall, and F1 score, were tracked.

**FIGURE 3:**
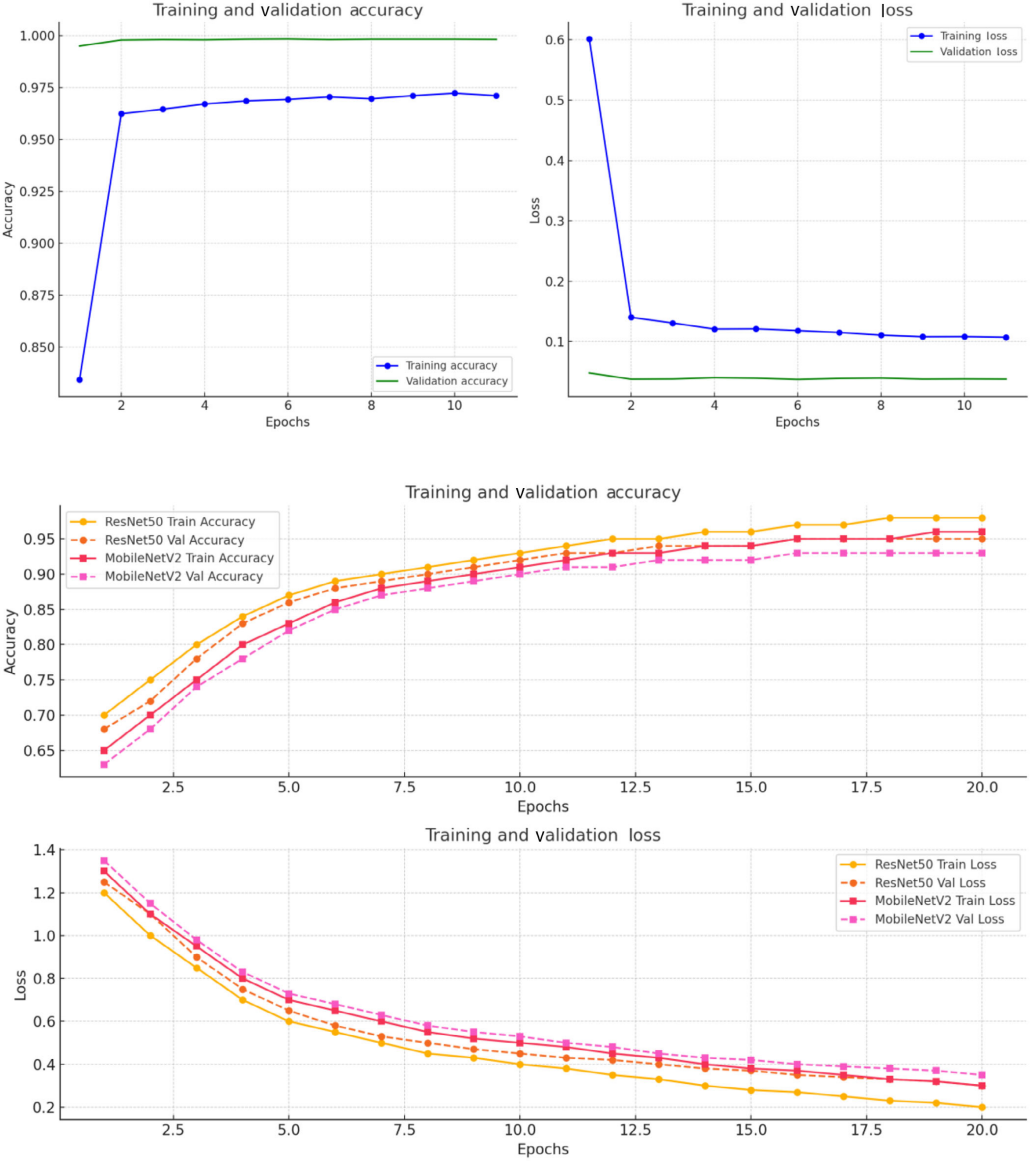
Training and validation accuracy and loss of hybrid long short‐term memory–feedforward neural network model and ResNet50 and MobileNetV2 models.

Early stopping terminated training at the 11th epoch of 20 when validation loss failed to improve for five consecutive epochs for the structured dataset, thereby preventing overfitting. See Figure 3 for training and validation detail of the hybrid LSTM‐FNN model used for the structured dataset.

### 2.11 Performance evaluation

**FIGURE 4:**
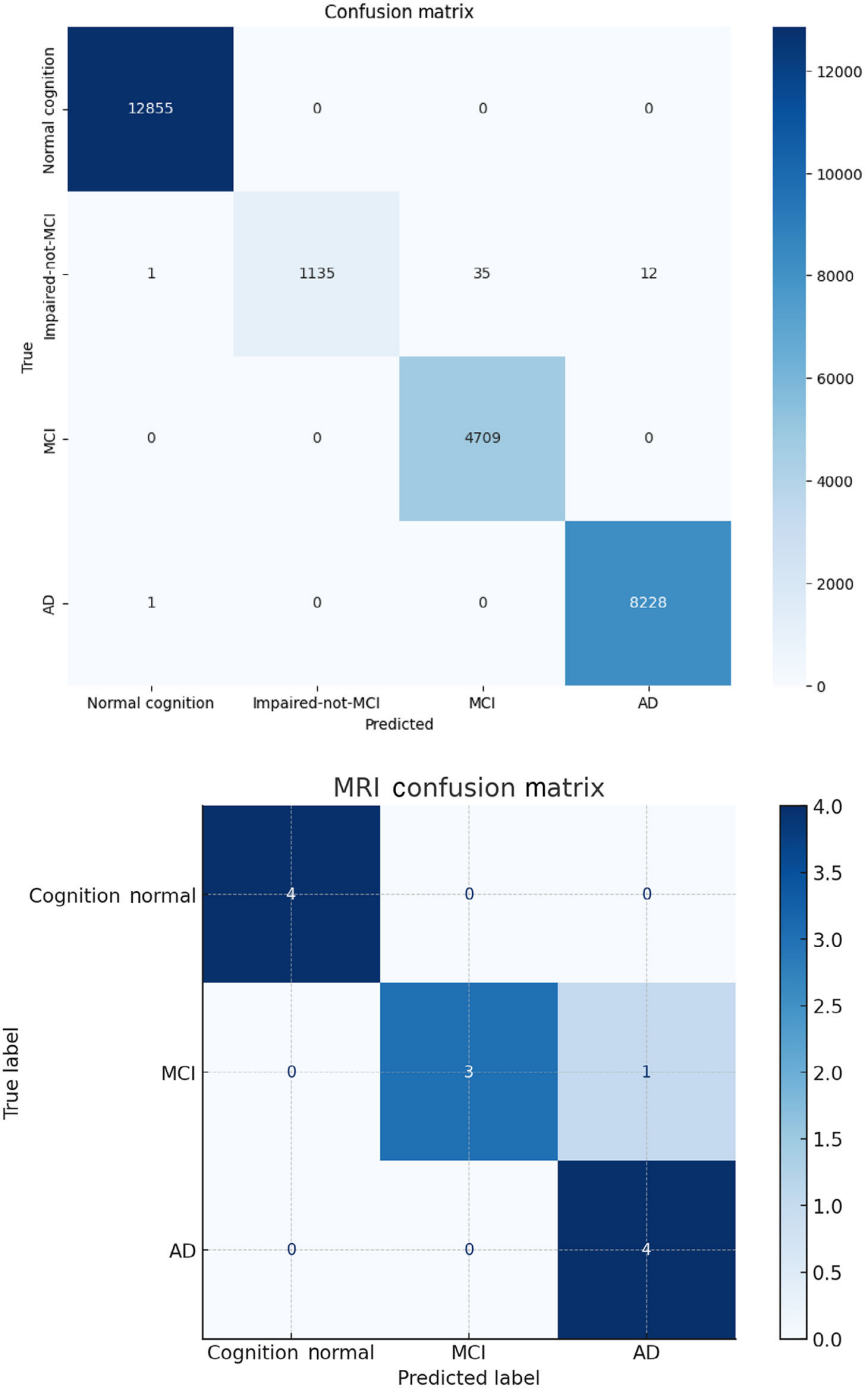
Correlation matrix of prediction results for hybrid long short‐term memory–feedforward neural network model and MRI‐based ResNet50 and MobileNetV2 models.

The structured and MRI‐based models were evaluated separately using accuracy, precision, recall, and F1 score. The independent evaluation of these models enables healthcare decision‐makers to compare their predictions and leverage complementary insights when integrating them into clinical workflows. Figure 4 shows how both models attempted to match the target labels and the mismatches across classes.

## 3 RESULTS

The development of independent models for structured data and MRI images represents a significant step forward in AD research, offering modular approaches to detecting disease progression. By separately analyzing temporal, static, and spatial patterns, the study captures the multifaceted nature of AD pathology while providing flexibility in the clinical application of these models.

### 3.1 Contributions of independent models

The design of the independent structured and MRI‐based models leverages the unique strengths of each data type.

#### 3.1.1 Structured data models

Clinical records and cognitive measures over time were captured as temporal dependencies using the LSTM model. While the FNN model processed non‐sequential features, ensuring distinct and non‐redundant patterns informed the predictions. The use of correlation‐based pruning minimized redundancy and allowed the model to focus on highly informative features.

#### 3.1.2 MRI‐based models

Using ResNet50 and MobileNetV2, the MRI‐based models extracted spatial patterns indicative of structural changes, such as cortical thinning and hippocampal atrophy, which are key determinant features of AD.

These models analyzed high‐level spatial patterns and subtle regional variations independently, showcasing their ability to address the unique aspects of AD symptoms present in the imaging data.

By independently evaluating the structured and MRI‐based models, this approach enables healthcare professionals and researchers to analyze predictions from each domain separately or integrate insights based on specific clinical needs. This method enhances interoperability and facilitates implementation across diverse healthcare settings.

### 3.2 Comparison with existing approaches

#### 3.2.1 NACC dataset comparison

Comparison of the hybrid LSTM‐FNN model and the SVM‐based approach^6^ underscores the advantages of deep learning in AD classification. Trained on the same NACC dataset, the hybrid LSTM‐FNN model achieved 99.82% accuracy, significantly surpassing the SVM model's 90.5% accuracy in multiclass classification (NC, MCI, AD). This demonstrates deep learning's ability to extract meaningful features from high‐dimensional data without manual feature engineering.

Beyond classification, the hybrid LSTM‐FNN model offers superior predictive capabilities. While the SVM‐based approach relied on rule‐based feature selection to predict AD progression (F1 score: 88% for binary, 72.8% for multiclass), the LSTM component of the hybrid model is inherently designed to capture longitudinal dependencies, making it better suited for time‐series forecasting and progressive AD detection.

Also, unlike the SVM model, which applied rules that excluded potentially valuable data, the hybrid LSTM‐FNN model fully leveraged the multimodal nature of the NACC dataset, integrating sequential and static features simultaneously. This adaptability enhances its real‐world application for early AD detection and prognosis. This also proves that the hybrid model combining the strength of LSTM and FNN provides a better result when also compared to standalone models, as observed in a first experiment we performed where a standalone LSTM model achieved 89.41% accuracy, 90.03% precision, 89.41% recall, and 88.49% F1 score. For the standalone FNN the following metrics were met: 89.46% accuracy, 89.81% precision, 89.46% recall, and 88.52% F1 score. These findings highlight the potential of deep learning models, particularly those incorporating sequential learning, for improved AD classification and progression analysis.

#### 3.2.2 ADNI dataset comparison

Comparison of the MRI‐based model utilizing ResNet50 and MobileNetV2 and the alternative deep learning approach by Nagarathna and Kusuma^7^ (both studies used ADNI dataset) highlights the advantages of advanced convolutional neural networks (CNNs) in AD classification. While the referenced study employed multiple models, including multilayer perceptron networks, random forest, SVM, and decision tree classifiers, achieving an accuracy of 95.52% and 91.25% of precision, recall, and F1 score, the ResNet50 and MobileNetV2 models offer superior feature extraction capabilities, leading to enhanced classification performance accuracy of 96.19%, 96.89% precision, 96.19% recall, and 96.34% F1 score.

ResNet50 and MobileNetV2 are optimized for image‐based learning, leveraging deep feature hierarchies and efficient parameter utilization. Compared to traditional ML models and fully connected neural networks, these architectures excel in capturing spatial patterns in MRI images, resulting in improved generalization and robustness. Additionally, while the referenced study incorporated clinical data alongside MRI images, its reliance on multiple classification models lacks the efficiency and consistency of a unified deep CNN approach.

The hybrid use of ResNet50 and MobileNetV2 allows for a balance between model accuracy and computational efficiency, making the approach more scalable for real‐world deployment. Unlike conventional deep learning models that require extensive manual feature selection, CNN‐based architectures autonomously learn discriminative features, minimizing preprocessing efforts.

The combined strengths of ResNet50 and MobileNetV2 also outperformed a standalone ResNet50 experiment performed on the same dataset with accuracy at a low of 68.51%, 64.21% precision, 65.21% recall, and 65.62% F1 score. Similarly, a standalone MobileNetV2 also underperformed at 53.12% accuracy, 52.45% precision, 52.20% recall, and 50.51% F1 score. These advantages position the combined ResNet50 and MobileNetV2‐based approach as a more advanced and practical solution for accurate AD classification and early detection.

The structured data models successfully captured temporal and static patterns, while the MRI‐based models leveraged pretrained architectures for robust spatial analysis. Both approaches achieved competitive performance when benchmarked against state‐of‐the‐art hybrid models by Alatrany et al.^6^ and Nagarathna and Kusuma.^7^ Table 2 shows a breakdown of the experiment results.

**TABLE 2:** Experimental results comparing different models on the ADNI MRI and NACC structured datasets based on accuracy, precision, recall, and F1‐score.

S/N	Models	Dataset used	Accuracy (%)	Precision (%)	Recall (%)	F1 score (%)
1	ResNet50 and MobileNetV2 MRI‐based model	ADNI MRI images	96.19	96.89	96.19	96.34
2	Hybrid LSTM‐FNN	NACC structured dataset	99.82	99.82	99.82	99.82
3	Standalone ResNet50 MRI‐based model	ADNI MRI images	68.51	64.21	65.21	65.62
4	Standalone MobileNetV2 MRI‐based model	ADNI MRI images	53.12	52.45	52.20	50.51
5	Standalone LSTM	NACC structured dataset	89.44	89.31	89.44	88.58
6	Standalone FNN	NACC structured dataset	89.50	90.08	89.50	88.29

Abbreviations: ADNI, Alzheimer's Disease Neuroimaging Initiative; FNN, feedforward neural network; LSTM, long short‐term memory; NACC, National Alzheimer's Coordinating Centre.

### 3.3 Strengths and advantages

By training and evaluating the models independently, this approach offers flexibility in clinical decision‐making. Each model can be deployed as a standalone tool or used in combination, depending on the availability of data and clinical requirements.

The systematic preprocessing pipeline ensured high‐quality input data. For structured data, methods like correlation‐based pruning reduced redundancy, while image augmentation techniques improved generalization in MRI‐based models.

The use of participant‐based data splitting ensured independence between training, validation, and testing datasets, mitigating overfitting and preventing data leakage, which can lead to inaccurate model performance due to the model seeing the same data used for testing and validation during the training stage.

**FIGURE 5:**
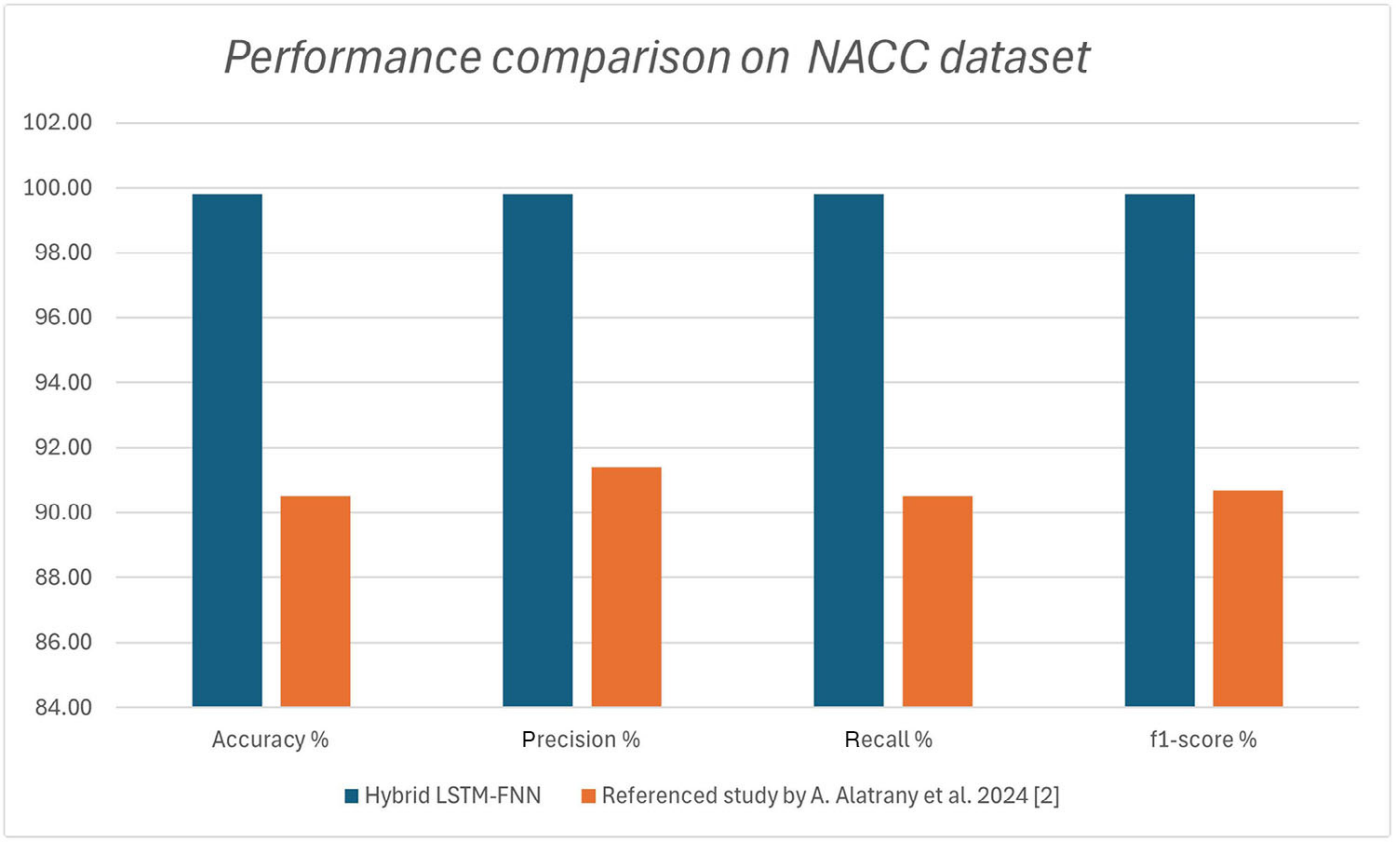
Performance comparison between proposed hybrid long short‐term memory–feedforward neural network model and reference support vector machine‐based model of Alatrany et al.^6^ on National Alzheimer's Coordinating Centre dataset, measured by accuracy, precision, recall, and F1‐score.

**FIGURE 6:**
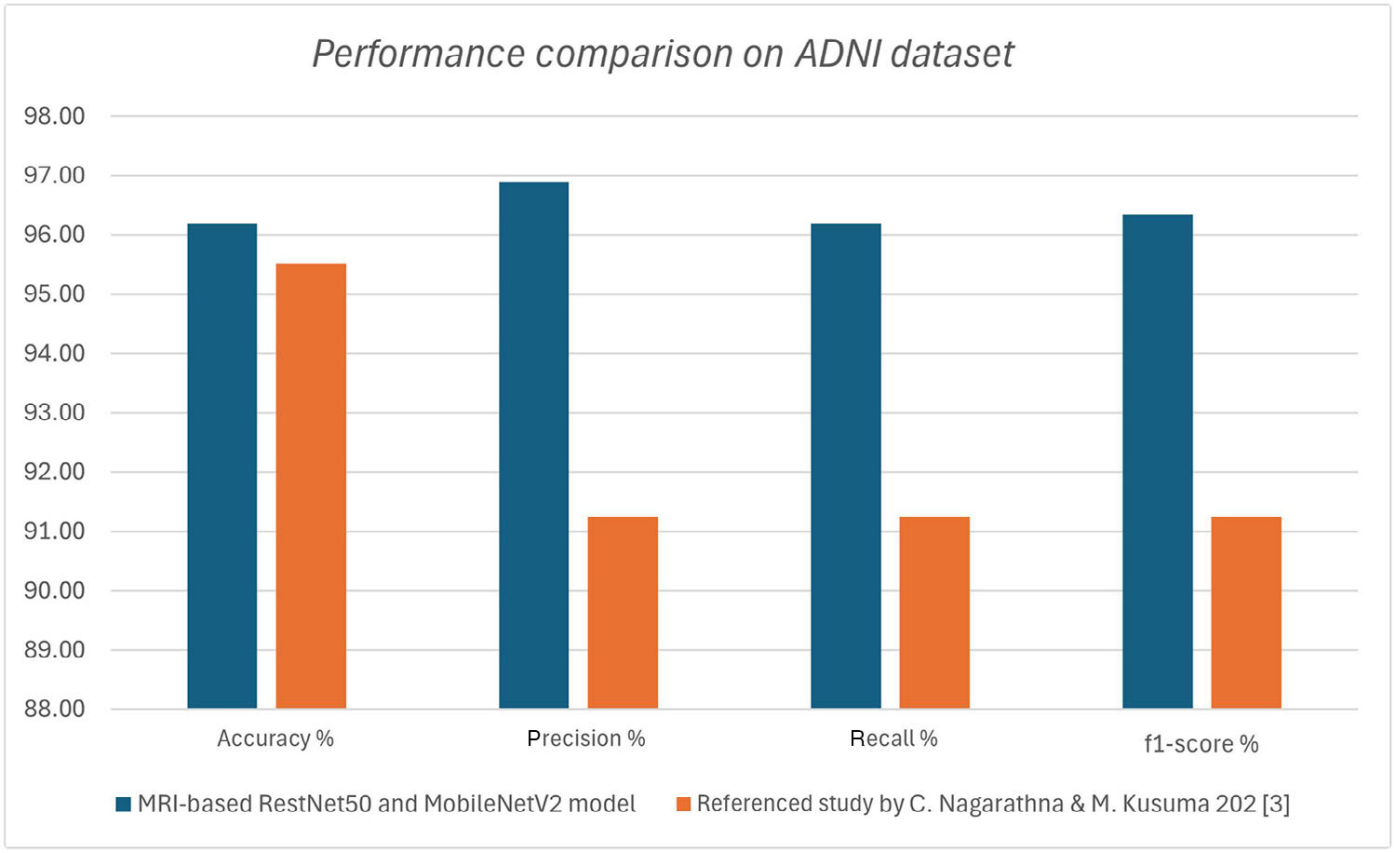
Performance comparison of proposed ResNet50 and MobileNetV2 models against reference traditional models by Nagarathna and Kusuma on Alzheimer's Disease Neuroimaging Initiative dataset.

The independent models can be easily integrated into existing healthcare workflows, allowing for tailored predictions based on available data types. Figures 5 and 6 present bar charts comparing the performance of the proposed models against their respective benchmarks on similar datasets.

### 3.4 Overfitting management

Several strategies were implemented to enhance the reliability and generalizability of the models. Participant‐based splitting ensured that data from the same individual was not shared across training, validation, and test sets, maintaining the independence of datasets.^19^ Dropout layers and the reduction of model complexity, such as lowering the number of LSTM units, further minimizing the risk of overfitting. The diversity in the NACC and ADNI datasets contributed to mitigating biases and improving generalizability, ensuring the models can perform well across varied populations and settings.

This study demonstrates the effectiveness of developing and evaluating independent models for structured data and MRI images. By addressing temporal, static, and spatial patterns independently, this approach provides a flexible, modular framework that can be adapted to diverse healthcare scenarios. Future work should focus on integrating these models into clinical decision systems and expanding their application across broader populations and modalities.

## 4 DISCUSSION

This research presents a two‐way approach to AD detection. The capabilities of structured data and MRI images on independently trained models are analyzed using independently trained deep learning models. Each data modality (i.e., the structured and image datasets) was used to train a model that could leverage the information in them, which is why the structured data were used for LSTM and FNNs to capture temporal dynamics and static feature correlations, respectively. On the flip side, the MRI‐based model uses pretrained architectures, ResNet50 and MobileNetV2, to identify high‐level spatial patterns and subtle regional changes indicative of AD progression.

The approach of two differently trained and evaluated models is critical for a conceptual healthcare system that can leverage either structured data such as questionnaires or medical images like MRI to support decision‐making in AD diagnosis. This means the system is capable of processing both or either one of the kinds of data available, thereby improving flexibility, scalability, interoperability, and easy integration into diverse clinical settings.

After rigorous testing and evaluation, both models demonstrated a high level of performance in their respective domains, highlighting their reliability and effectiveness for early AD detection.

### 4.1 Future directions

AD, like all critical healthcare challenges, allows no margin for error. Therefore, it is important to conceptualize and discuss possible future improvements to the models developed in this research, regardless of the acheived performance. The next key step is to build the conceptualized system where these models will function as a comprehensive clinical decision system. By comparing or integrating predictions from these independent models, clinicians could benefit from a holistic evaluation tool that provides a view of a patient's condition, enabling more precise and personalized diagnostic insights. Such a system will enable healthcare professionals to focus on critical activities such as preparing accurate cognitive tests, experimenting on biomarkers, and producing MRI images that the system can analyze and produce interpretable outcomes for final decisions. This could streamline workflows and aid in early intervention strategies.

Further evaluation studies are required to widen the success net of these models. Datasets that cut across other geographical areas and demographically diverse cohorts, specifically cohorts of younger age groups, are critical to this improvement. This is crucial as AD exhibits different symptoms across populations due to genetic, environmental, and lifestyle factors. More data from younger cohorts are also crucial to early diagnosis, current symptoms are predominantly evident in older cohorts; hence, monitoring younger cohorts will further increase the ability of models to understand what changes contribute to this disease from an early age. These factors will further improve models’ generalizability and ensure that they will perform effectively across varied patient groups, minimizing potential biases and broadening their global utility.

Another critical area of development is the implementation of uncertainty estimation methods within the models. Confidence scores or probability estimates accompanying predictions could enhance their reliability in clinical practice by helping practitioners understand the degree of certainty associated with each prediction. This feature is especially vital in high‐stakes decisions, where understanding the model's confidence can guide further investigations or second opinions.

If models like these are to be deployed in rural settings, further computational optimization techniques such as model pruning or lightweight architecture must be explored. This will reduce resource demand without sacrificing model performance.

Extending the models for real‐time prediction opens exciting possibilities, particularly for monitoring AD progression over time. Real‐time analysis of structured data, such as cognitive scores or biomarker levels, and live imaging could allow for continuous monitoring of patients, providing clinicians with dynamic insights and enabling timely interventions. This capability would mark a significant shift in how AD is managed, moving from manual assessments to proactive and ongoing care.

By focusing on independently trained and evaluated models, this approach sets the stage for adaptable and scalable diagnostic systems. Future efforts to refine these models and expand their applications promise to enhance early detection, improve patient outcomes, and pave the way for more effective and inclusive strategies in combating AD.

## CONFLICT OF INTEREST STATEMENT

The authors declare no conflicts of interest. Author disclosures are available in the supporting information.

## CONSENT STATEMENT

Not applicable.

## Supporting information



Supporting Information
